# A Self-Regulation Theory–Based Asthma Management Mobile App for Adolescents: A Usability Assessment

**DOI:** 10.2196/humanfactors.7133

**Published:** 2017-02-01

**Authors:** Adam Sage, Courtney Roberts, Lorie Geryk, Betsy Sleath, Deborah Tate, Delesha Carpenter

**Affiliations:** ^1^ Division of Pharmaceutical Outcomes and Policy Eshelman School of Pharmacy University of North Carolina at Chapel Hill Chapel Hill, NC United States; ^2^ Department of Nutrition Gillings School of Global Public Health University of North Carolina at Chapel Hill Chapel Hill, NC United States; ^3^ Department of Health Behavior Gillings School of Global Public Health University of North Carolina at Chapel Hill Chapel Hill, NC United States

**Keywords:** mHealth, asthma, mobile, usability

## Abstract

**Background:**

Self-regulation theory suggests people learn to influence their own behavior through self-monitoring, goal-setting, feedback, self-reward, and self-instruction, all of which smartphones are now capable of facilitating. Several mobile apps exist to manage asthma; however, little evidence exists about whether these apps employ user-centered design processes that adhere to government usability guidelines for mobile apps.

**Objective:**

Building upon a previous study that documented adolescent preferences for an asthma self-management app, we employed a user-centered approach to assess the usability of a high-fidelity wireframe for an asthma self-management app intended for use by adolescents with persistent asthma.

**Methods:**

Individual interviews were conducted with adolescents (ages 11-18 years) with persistent asthma who owned a smartphone (N=8). Adolescents were asked to evaluate a PDF app wireframe consisting of 76 screen shots displaying app features, including log in and home screen, profile setup, settings and info, self-management features, and graphical displays for charting asthma control and medication. Preferences, comments, and suggestions for each set of screen shots were assessed using the audio-recorded interviews. Two coders reached consensus on adolescent evaluations of the following aspects of app features: (1) usability, (2) behavioral intentions to use, (3) confusing aspects, and (4) suggestions for improvement.

**Results:**

The app wireframe was generally well received, and several suggestions for improvement were recorded. Suggestions included increased customization of charts and notifications, reminders, and alerts. Participants preferred longitudinal data about asthma control and medication use to be displayed using line graphs. All participants reported that they would find an asthma management app like the one depicted in the wireframe useful for managing their asthma.

**Conclusions:**

Early stage usability tests guided by government usability guidelines (usability.gov) revealed areas for improvement for an asthma self-management app for adolescents. Addressing these areas will be critical to developing an engaging and effective asthma self-management app that is capable of improving adolescent asthma outcomes.

## Introduction

Asthma is the most common chronic condition among youth in the United States, affecting 8.6% of children under the age of 18 years (10.1% of males and 7.0% of females) [1]. Prevalence rates in youth are higher than those for adults, which are 7.4% overall (5.1% of males and 9.6% of females) [1]. The negative impacts of asthma for youth include decreased quality of life [2], nearly 10 million missed days of school a year, a half million emergency department visits annually [3], and limited ability to engage in normal daily activities, such as taking part in physical activities (eg, sports and exercise) and other outdoor activities and extracurriculars [4]. For many adolescents, self-management behaviors such as medication adherence, trigger avoidance, monitoring symptoms, and communication with health care providers and family members can prevent or reduce the negative impacts of asthma [5-7]. Unfortunately, patient-provider communication about self-management behaviors among adolescents is often inadequate [8-11], which may partially explain why adherence to asthma controller medication is low (50%-70%) for adolescents [12-14].

**Figure 1:**
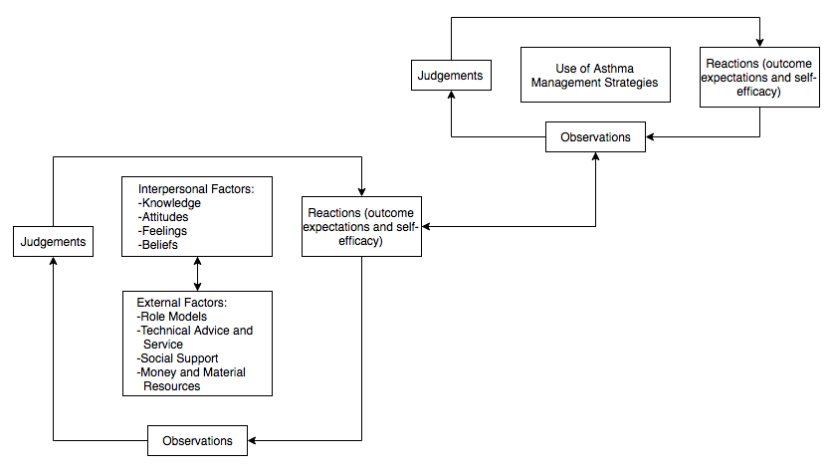
The self-regulation process as it relates to use of asthma self-management strategies [15].

Self-regulation theory (SRT) posits that one possesses the ability to influence his or her own behavior by being observant, making judgements about behavior, and reacting accordingly based on those observations and judgments [15]. This process (presented in Figure 1) can be achieved in several ways, including through self-monitoring one’s own behavior and behavioral feedback or information about a task intended to improve performance [15]. Smartphones are now capable of facilitating self-regulating health behaviors, and mobile-based interventions are increasingly capable of addressing barriers to medication adherence [16]. In fact, several mobile apps exist to manage asthma, which is important since text messaging is a preferred method for communicating asthma information among 12 to 17-year-olds [17]. Unfortunately, although smartphone adoption rates for teens aged 13 to 17 years are on the rise (73%) [18], only 8 of 147 (5.4%) existing asthma apps target children or young adults [19]. Furthermore, to our knowledge, the theoretical pathways through which existing asthma apps operate to influence self-management behaviors have only been reported in 1 study [20,21]. A 2013 Cochrane Review located only 2 randomized controlled trials (RCTs) that tested the effects of asthma self-management apps. However, these RCTs did not link app features to asthma outcomes, which led the authors to suggest that future apps should have theory-based features and study designs that allow researchers to identify which components (ie, app features) of the intervention are effective [22].

According to Usability.gov, benefits of a user-centered design for mobile apps include improved performance (eg, fewer user errors) and credibility (eg, user satisfaction and trust of the app) [23]. These benefits are particularly important when it comes to managing asthma symptoms, triggers, and medication. The approach to app development used in this study adheres to mobile app development and design guidelines outlined by Usability.gov, which provides guidance for ensuring mobile apps are useful, usable, desirable, and accessible and consist of content relevant to the user that is also credible [23]. Usability tests are an important part of determining if such guidelines are met throughout the app development process. This study is a formative test of an app wireframe, which is an important step in the usability lifecycle [24]. Results from this study will guide subsequent efforts to develop an app for adolescents that optimizes performance for the user while providing an experience that both engages and encourages asthma self-management.

In a previous study, we examined the theoretical pathways through which asthma management apps promoted self-management for adolescents [20,21]. Specifically, we asked adolescents (n=20) aged 12 to 16 years to use 2 existing asthma self-management apps and conducted semistructured interviews to identify specific app features that promoted self-observation, self-judgment, and self-reaction (key components of SRT). Our findings identified several potentially useful app features that align with key components of SRT, including features that promote self-observation and self-judgement (monitoring symptoms, triggers, and medication) and features that promote self-reaction (viewing charts based on data from logging medication adherence, symptoms, and triggers and asthma control quizzes). Results were used to inform the development of a high-fidelity asthma app wireframe described here.

The purpose of this study is to assess the usability and user-centeredness of a high-fidelity wireframe for an asthma self-management app intended for use by adolescents with persistent asthma. We employed a user-centered approach [23] in developing the wireframe by conducting interviews with adolescents to test its usability, including usability of specific self-management features such as logging medication and setting medication reminders. To do this, we posited several research questions to ascertain the visual aspects of design (Do participants like or dislike how the app looks and feels?), the app’s intended functionality (Do participants understand the desired functionality of different app features?), areas for improvement (Are there any changes participants would make to the app?), expectations of the app (Does the app appear to perform the tasks they would expect from an asthma self-management app?), and behavioral intentions related to app features (How do participants anticipate using the app and its specific features?). These data can be used to guide the development of adolescent self-management features that are theory-based, user-centered, and perceived as useful by adolescents.

## Methods

### Participant Recruitment

A purposive sample of 8 adolescents was recruited from 3 pediatric medical practices in North Carolina and via word of mouth. Adolescents were sampled to maximize racial/ethnic, age, and gender diversity. Eligible participants were between the ages of 11 and 18 years, spoke and read English, had a self-reported diagnosis of persistent asthma, and owned either a smartphone or tablet. Parents or guardians provided written consent, and adolescents provided written assent.

### Data Collection Procedure

A research assistant (AS) trained in usability test methods conducted all 8 in-person exploratory usability tests using semistructured interviews [24,25]. Each interview lasted approximately 45 minutes. Participants first verbally answered demographic questions as well as general questions about mobile app and Internet use. Participants were then presented with an electronic PDF wireframe of a mobile app for managing asthma. The wireframe consisted of 76 screenshots representing different sections of the app and different app features, including log-in and home screen (2 screens); profile set-up (17 screens); settings and app information (4 screens); an asthma control quiz (5 screens); gamification (quizzes and badges) (10 screens); logging medications, symptoms, and triggers (19 screens); charting (7 screens and 2 separate printed charts); and notifications, reminders, and alerts (11 screens). A detailed description of each feature, the number of screenshots for each feature, and the associated questions and measures for each feature are presented in Multimedia Appendix 1. Example screenshots of each feature shown to participants are also included in Multimedia Appendix 1. Following a complete review of the wireframe, summary questions were asked. Adolescents received a $25 gift card incentive upon completion. The research protocol for this study was reviewed and approved by the institutional review board at the University of North Carolina at Chapel Hill.

### Measures

Prior to commencing the usability tests, participants were asked their age in years, gender, race (white, black, other–specified) and ethnicity (Hispanic, non-Hispanic), how many hours they use the Internet per week (open-ended), and how many hours they use mobile apps per week (open-ended).

An interview protocol was developed to guide the usability tests [24,25]; the questions are presented in Multimedia Appendix 1. Participants were asked to evaluate several aspects of the app: whether they liked or disliked features (yes/no), what they liked or disliked about the features (open-ended), if there was anything about the feature that was confusing or missing (yes/no), if there is anything they would do differently to improve the feature (open-ended), and if customization (ie, allowing user-specific changes to the app) was mentioned (yes/no). Several open-ended questions were also used to obtain user input about the look and feel of the log-in and home screen (eg, colors, logos, and organization), how often they would take an asthma control quiz, and how they would like to be reminded to use medications (eg, regularly or only when missed). Participants were also encouraged to provide any additional feedback not specifically solicited by the interviewer.

Participant preferences for information visualizations within the charting feature were also assessed. Specifically, participants were asked to review 2 types of charts: (1) a line graph plotting asthma control over time with dots indicating logged triggers, symptoms, and medication adherence and (2) a bar chart using only dots to show the same information. Each type of visualization showed identical information about asthma control, controller medication adherence, symptoms, and triggers over a 7-day period. Participants were asked to rate the asthma control of the person represented in the charts on a 4-point scale (1=very uncontrolled to 4=very controlled). Participants were then asked to choose which of the 2 charts they would prefer to use to see their own data.

Finally, after having reviewed the entire wireframe, participants were asked several summary questions about the app. These included open-ended questions such as overall likes and dislikes and the top 3 things participants liked and disliked about the app. Participants were also asked to rate their likelihood of using the app to manage their asthma on a 5-point scale (1=not at all likely to 5=very likely).

### Data Analysis

Interviews were audiorecorded for coding and analysis. Unique IDs were assigned to each participant to deidentify responses. Two independent coders (AS and CR) listened to the audiorecorded interviews and coded responses for each section or set of screenshots representing different app features. The first coder (CR) coded all 8 interviews, while the second coder (AS) coded 4 randomly selected interviews. Multimedia Appendix 1 summarizes how data for each feature were coded (yes/no, scale, or open-ended). Whether a participant liked or disliked a feature was coded as yes/no, and questions soliciting open-ended responses were transcribed verbatim and assessed for common themes.

To assess interrater reliability, separate Cohen kappa coefficients were calculated using a random selection of 50% of coded items for each feature, questions on previous app and Internet usage, and summary questions. Interrater reliability scores ranged from good (.70-.90) to very good (.90-1.0).

## Results

### Sample Characteristics and Adolescent Technology Use

Table 1 summarizes the demographic characteristics and self-reported technology use of the study participants. The sample included 4 males and 4 females, with an average age of 14.2 years; 4 participants were white, 3 were black, and 1 was Hispanic. Participants reported using the Internet on any device an average of 26 hours per week and reported using mobile apps (on a smartphone or tablet) an average of 24 hours per week. The types of mobile apps participants reported using most were games (n=5) and social media (n=3).

**Table 1:** Sample characteristics.

Characteristics		N=8
Age, years, mean (SD)		14.2 (2.5)
Male, n (%)		50 (4)
**Race/ethnicity, n (%)**		
	Non-Hispanic White	50 (4)
	Non-Hispanic Black	38 (3)
	Hispanic	13 (1)
Hours using the Internet per week, n, median, range		26, 19, 2-70
Hours using mobile apps per week, n, median, range		24, 12, 3-60

### Initial Impressions

All participants liked the overall look and feel of the app citing the colors and that it looked clean and professional.

I really like the design of it; I like the color scheme; it looks real clean. Not too much busy-ness going on.

Female, white, 15 years

It is appealing to the eye.

Male, black, 15 years

### Profile

The profile consisted of 17 screens, which demonstrated steps for setting up the profile: (1) uploading an asthma action plan document, (2) adding medications (type and dosage), (3) adding allergies and triggers, (4) setting goals, (5) creating an avatar, and (6) adding and editing personal information and emergency contacts. Half of the participants (4/8) mentioned a desire for customization of certain aspects of the profile, such as the option to manually enter medications, symptoms, triggers, and goals that are not included on existing dropdown lists.

Of the 8 participants, 5 liked the idea of having an avatar, although a few were less enthusiastic. This did not differ by age.

If [the avatar] is going to teach me about asthma, I don't really care.

Male, white, 12 years

### Settings and Information

A total of 4 screens showed settings and information for the app, which displayed where additional educational information could be found (eg, video tutorials and informational websites), as well as notification on/off buttons and volume controls. When asked if something were missing or if they would do something differently, only 1 participant suggested a change, citing it would be useful to have an in-app search function that directed to Internet resources rather than just listing links to informational websites. No participants found the settings or educational information confusing.

Participants were asked to indicate whether providing information (eg, links or videos) on 7 different topics would be useful to include in the app. Participants found the following useful: how medications work in the body (8/8), how to avoid triggers (8/8), how to tell the difference between a rescue inhaler and control medication (7/8), how to tell when your asthma is not well controlled (7/8), how to talk with your doctor about your medication (5/8), how to use your inhaler (4/8), and how to remember to take your medication (3/8).

### Gamification

The wireframe components included a gaming feature consisting of 11 screens, which presented a mock asthma knowledge quiz and badges awarded for (1) scoring well on the quiz, (2) adhering to medication, (3) consistently logging medication, (4) consistently logging symptoms, (5) consistently logging triggers, and (6) having well-controlled asthma. Overall, the idea was well received with 7 of 8 saying they liked the idea of games and 5 of 8 liking the idea of badges. However, open-ended feedback was not very enthusiastic. A participant stated a badge seemed interesting “but you can't use it towards anything” [Female, white, 11 years].

### Asthma Control Quiz

The asthma control quiz showed 5 screens with 3 example questions. The example questions displayed the question text (eg, How is your asthma today?) with the following response options (very good, good, bad, very bad). Corresponding emoji faces were also depicted along with each response option. A total of 5 of 8 participants indicated that an asthma control quiz would be useful. Participants indicated they would engage more with an asthma control quiz if it were shorter and more accessible. A participant stated that they would use a quiz “if it was kind of short, maybe every day, maybe every week, maybe every few days” [Female, white, 11 years]. In response to the emoji faces, participants were accepting of them but did not see them as necessary.

[The faces are] not absolutely necessary but I guess they help.

Female, white, 11 years

I don't think they're necessary but I think they're cute.

Female, white, 15 years

### Logging Medications, Symptoms, and Triggers

There were a total of 19 logging screens, which detailed the processes for logging medication use, symptoms, and triggers. The logging feature was generally well received. A participant found the logging feature useful “because I usually forget stuff like that” [Female, Hispanic, 17 years]. Participants found using and navigating the logging feature to be intuitive, but 4 of 8 participants commented that some form of customization would improve the feature. A common suggestion for customization included adding one’s own symptoms and triggers (ie, not from a dropdown list). A participant offered a suggestion that might improve engagement, citing she would like the app to “go more quickly” [Female, white, 15 years] by making the logging process more simple because logging information is not fun, and that in doing so more people might use the app.

### Charting

Participants were presented with 7 screens that depicted the progression through the charting feature. These screens included menu items to view information about the user’s asthma control, medication adherence, symptoms, and triggers, followed by a chart with all information over a 1-week period in a single visualization. Feedback from participants about the charting feature was generally positive.

A lot of people are visual learners. . . they will understand things better.

Male, black, 14 years

Multimedia Appendix 1 presents the 2 chart types presented to participants. Interestingly, all participants rated the bar/dotted chart as more controlled than the line graph for asthma control despite them depicting the same level of control. When asked which data visualization they preferred (line graph vs bar chart), 7 of 8 participants preferred line graphs. Feedback suggested that charting information longitudinally is appropriate for the target user age group (adolescents).

I prefer seeing the graph, honestly. If you could have a line graph for every one of them, that would be my preference, because it's easier to kind of watch how it goes up and down. . . It's really easy to see visually what's going on.

Female, white, 15 years

I like [the line graph] better because it shows throughout the week how it's progressed over time.

Male, black, 15 years

### Notification, Reminders, and Alerts

Participants were shown 11 screens related to notifications, reminders, and alerts for medication (medication reminders and notifications of missed doses), doctor’s appointments (reminders set in a calendar view), and triggers (alerts and notifications). All 8 participants said they would use the feature to remind them to use their inhaler, 7 of 8 participants said they would use the feature to alert them of triggers, and 6 of 8 said they would use the feature for doctor’s appointment reminders. An older participant (aged 18 years) pointed out the doctor’s appointment reminder would be useful.

So let's say you go out of town, right? Let's say you give your medication to like someone in your family so they can hold on to it. And then you forget about it, that you even have it with you. You have a reminder that tells you 'hey don't forget to take your medication.’

Male, black, 15 years

I think the alerts are good because I want this app to alert me when to take my medication.

Female, Hispanic, 17 years

I like the reminders. I use my calendar a lot, so it's nice just for doctor’s appointments and medications and stuff like that. So yeah, I would use it. . .often.

Female, black, 18 years

Feedback about how and when reminders, notifications, and alerts should be delivered indicated that these features should be customized to individual users. Smartphones have several options for delivering notifications, including sound, vibrations, and visual cues, and they have even more specialized settings (eg, types of sounds, banner notifications, and text message notifications) for each type of notification. Furthermore, the rules dictating when a notification, reminder, or alert is sent can vary (eg, every day or when a dose is missed). Customization was mentioned by 7 of 8 participants, so it appears a one-size-fits-all approach may not be ideal for optimal user engagement.

I want it to pop up on the screen like a text message.

Female, Hispanic, 17 years

I might get a little annoyed at the notifications.Male, white, 12 years]

Male, white, 12 years

### Final Questions

Following the review of the wireframe, participants were asked what they liked and did not like about the app. Feedback was generally positive.

I like how it was user friendly, looks professional, stuff like that.

Male, black, 14 years

It seemed pretty organized, which is, I like that a lot.

Female, Hispanic, 17 years

All 8 participants said they would find an app like this useful and reported they would be likely to use the app (mean 4.06, SD 18). Among the top 3 things participants liked about the app were notifications/reminders/alerts (5/8), quizzes and badges (5/8), charts (4/8), logging medications (3/8), and tracking triggers (3/8). Among the top 3 things participants did not like about the app were the bar charts (2/8), lack of customization (2/8), some of the labeling in the app (eg, specific graphics or icons) (1/8), and games (1/8). Some participants (3/8) did not designate any dislikes about the app.

## Discussion

### Principal Findings

To our knowledge, few asthma self-management apps exist that target adolescents [19], although ongoing studies address this research gap including the MyAirCoach project [26] and the CompAir trial [27], which seek to develop mobile-based asthma education and self-management technologies. This project seeks to do the same using a theory-driven approach. To our knowledge, no currently publicly available apps are based on any health behavior theory. This study is an important step in addressing this gap. In a previous study, we examined the theoretical pathways through which an asthma management app for adolescents is capable of improving self-management behaviors [20], which allowed us to assess the needs and requirements for the app and its features and incorporate them into a high-fidelity app wireframe. In this study, we examined the usability of the self-management features based on SRT and solicited feedback on the visual appearance and overall impressions of a wireframe of the app. The results from the usability tests provide an important understanding of how users expect to interact with an asthma management app, as well as their preferences while doing so. Specifically, our results suggest that users prefer the ability to customize a wide range of features including charting, notifications, reminders, and alerts.

In a previous study, a mobile asthma management app was shown to improve asthma control [28]. When surveyed postintervention, patients reported that the app was easy to use, relevant and personalized to their asthma, and provided helpful asthma-related information. By obtaining user feedback on our wireframe early in the development process, we believe we have identified key ways to integrate user-centered design into asthma self-management features to further increase an app’s ability to prompt better behavioral outcomes such as medication adherence, that can, in turn, lead to better asthma control. In our study, overall feedback regarding the look and feel of the app was positive. In particular, participants reacted positively to the aesthetics, including the colors, logos, icons, and organization. After the adolescent has decided to engage with the app, it then becomes important not to overlook aspects of the app that are not essential for carrying out the primary functions of the app but can improve or impede engagement. For instance, profiles are an important mechanism for customizing the user experience, and avatars, games, and badges may provide additional incentives to use the app. While not necessary for the app to function and complete necessary asthma management tasks (eg, logging medications or delivering cues to action), they may provide enhancements to the primary functions of the app in ways that promote engagement.

Promoting continuous engagement with a health app can be difficult, particularly with adolescents. Gamification is one way to promote engagement [29]. For example, rewarding positive behaviors (eg, medication adherence and regular logging of medication, symptoms, and triggers) with badges or trophies, improving asthma knowledge through quizzes, and unlocking avatar customization features (eg, dressing and accessorizing an avatar) as a reward for consistent app use are possible ways in which games or gamification can promote app engagement. However, where some features such as games and quizzes might be optional approaches to increase engagement, other necessary features require a certain level of effort by the user, which could negatively impact engagement. App developers should be cognizant of this. For instance, asthma control quizzes and logging medications, symptoms, and triggers are necessary tasks for self-managing one’s asthma, but the burden of doing so should be minimized. This can be accomplished in several ways, including minimizing the number of questions to accurately determine asthma control, allowing easy access to certain features (ie, limiting the extent to which drilling down into the app is required to reach certain features), and reducing the number of steps needed to complete tasks (eg, logging medication).

The primary focus of the usability tests was ascertaining feedback on the self-management features themselves, their functions, and how likely users envision incorporating them into their own asthma self-management behaviors. Of all the features, the charting feature and the notifications, reminders, and alerts feature received the most feedback, which was generally positive. However, there were also important suggestions that may improve the app’s usability. Like usability guidelines would suggest [23], we found that adolescents preferred a more personalized experience by customizing many features of the app. Both the charting feature and notifications, reminders, and alerts feature should provide a level of customization that both accomplishes its intended purpose (eg, providing visual feedback through patient data visualizations, or providing cues to action) without causing undue burden on the user.

For charting health data, customization might take the form of allowing individual charts and graphs (eg, separate charts for medication adherence, asthma control, symptoms, and triggers) as well as charts and graphs summarizing all logged information, allowing for custom colors and custom periods of time (eg, weekly vs monthly views). Developing a useful charting feature is important because charting improves self-judgement, which is key to promoting asthma self-management [20]. In our study, almost all participants preferred line graphs to display longitudinal information for asthma control, medication adherence, symptoms, and triggers. This finding is what we would expect considering common data visualization standards [30] and suggests that adhering to these standards and reaffirming the appropriateness of their application in communicating health data to a patient is important for ensuring optimal usability for mHealth applications. When designing such data visualizations, it is important not to burden the user with too much visual information in 1 chart or graph, as this may limit the comprehension of the information that is being communicated [31]. To address this, app designers should seek creative solutions that allow the user to comprehend information while adhering to data visualization standards [30].

The customization of notifications, reminders, and alerts can also take many forms. For instance, users might choose to be notified daily to take their medication or only when a dose is missed. Individuals can be prompted by notification when medication was not logged for the day. The frequency of notifications and how they are displayed is also customizable. For example, reminders can be linked to the native iPhone reminders or calendar app, sounds can be turned on and off, notification indicators can appear on the app icon and/or inside of the app itself, and notifications can be shown as banners or alerts that can either be dismissed or in the form of a prompt allowing the user to access the app from the notification itself. Trigger alerts can also be set by linking environmental triggers from weather alerts (eg, pollen, dust, smoke/fire, and temperature) to the app and can further be customized by location tracking.

### Limitations

This research is not without limitations. A convenience sampling method was used to purposively sample adolescents in order to ensure representation from males, females, different adolescent ages, and racially and ethnically diverse participants; however, these adolescents may not be representative of the broader population of adolescents with asthma. For the purpose of this study and the stage in the app development process where a high-fidelity wireframe was used, the sampling method and interview process was sufficient to obtain data on perceived usability of app features. Results from these assessments will be incorporated into a functional app, allowing for more summative usability testing in the future [24]. Given the one-on-one informal atmosphere of the usability tests, it is possible participants provided socially desirable responses. In an effort to prevent this, we told participants that we understand there may be things about the app they may not like and may find confusing. We described that the purpose of the interview was to obtain their feedback on the app wireframe, including what could be improved. We encouraged them to speak openly and honestly about the app. However, while several features or sections of the wireframe, including the profile, games, badges, settings and information, and logging medications, symptoms, and triggers received positive feedback overall, feedback on how to improve the app was limited. This may be a result of the format of the wireframe (PDF), and a functional prototype may have allowed users to understand the features and the tasks related to each feature better. For instance, the PDF wireframe used in this study presented several screenshots for logging medications, which allowed the participants to see the steps in the process. However, getting a true sense for the level of effort (eg, number of screen taps to complete a task) is limited by using a wireframe not capable of interaction.

### Conclusion

Our study is an important step in developing a useful and useable asthma management app for adolescents. Adhering to usability guidelines and ensuring adequate and appropriate usability testing throughout all stages of the app development cycle is important, especially when theoretical concepts of behavior change are integrated into a design in novel ways. The results from this study will be incorporated into a functional app intended to significantly improve asthma self-management for adolescents. A fully functional app will enable us to assess usability using quantitative experimental methods and sampling techniques that allow for a more representative sample of the target patient population (ie, all adolescents with persistent asthma owning smartphones or tablets). Finally, a fully functional app would provide a necessary intervention tool that would allow for comparisons of the effectiveness of existing asthma self-management apps and the theory-based and user-centered asthma self-management app currently being developed.

## Appendix

Multimedia Appendix 1 Semistructured interview questions and example screenshots used during usability tests.

## Abbreviations

RCT: randomized controlled trial
SRT: self-regulation theory
