# Supplementary material for: A Self-Regulation Theory–Based Asthma Management Mobile App for Adolescents: A Usability Assessment
Source: JMIR Hum Factors. 2017 Feb 1;4(1):e5. doi: 10.2196/humanfactors.7133 (PMC5311420; doi:10.2196/humanfactors.7133)
Supplement: Multimedia Appendix 1 [file humanfactors_v4i1e5_app1.pdf]

Appendix 1. Semi-structured interview questions and example screenshots used during usability tests

Feature	Number of Screens in Wireframe	Interview Items	Response option
Login/Home Screen	2	Do you like the overall look and feel?	Yes/No
		Is there anything you like or dislike?	Open-ended
		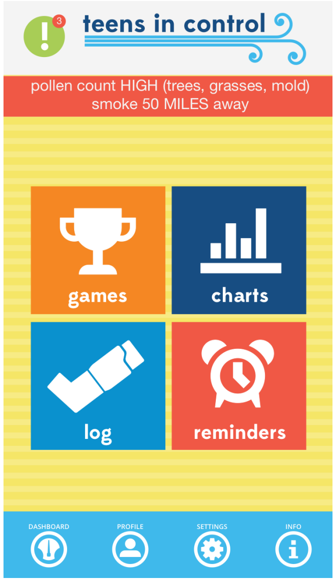	
Profile	17	Would having a profile be useful?	Yes/No
		Do you like the idea of having an avatar?	Yes/No
		Anything you expected to do but couldn't?	Open-ended
		Is there anything missing?	Open-ended
		Is there anything you would do differently?	Open-ended
		Is there anything about the profile set-up that is confusing?	Open-ended
		Mentions customization for inputting goals, medications, symptoms, or triggers	Yes/No

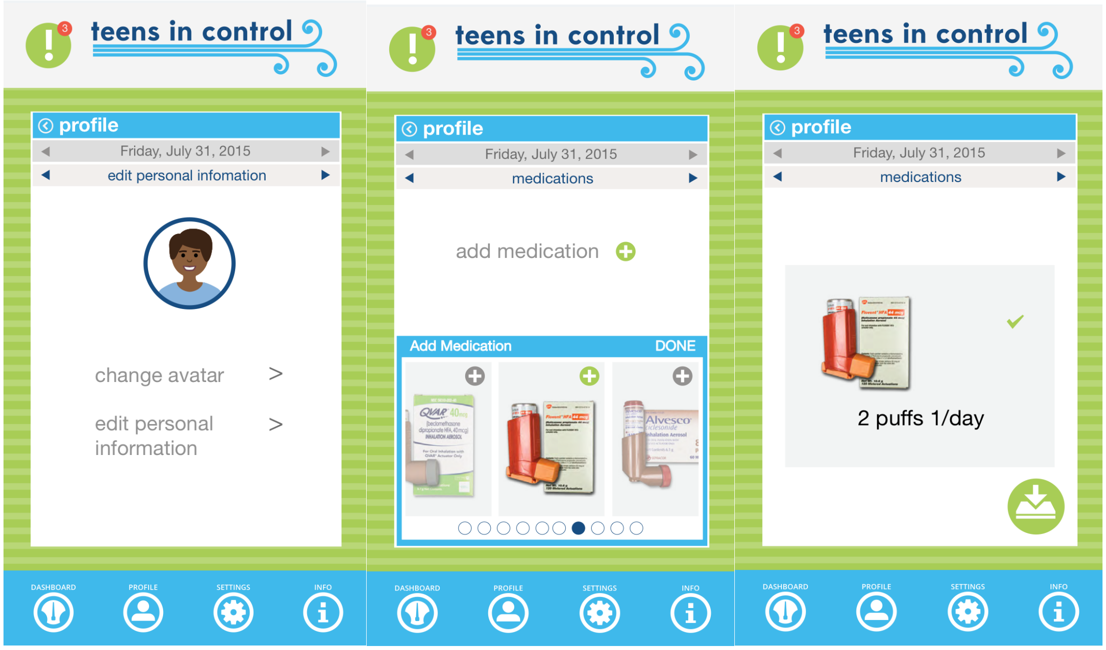

Settings and Info	4	Is there anything you like or dislike?	Open-ended
		Is there anything missing?	Open-ended
		Is there anything you would do differently?	Open-ended
		Is there anything confusing?	Open-ended
		Would it be useful for the app to include links to websites with information on:	
		How to use your inhaler?	Yes/No
		How your medications work in your body?	Yes/No
		How to tell the difference between a rescue and control medication?	Yes/No
		How to remember to take your medications?	Yes/No
		How to talk with your doctor about your asthma?	Yes/No
		How to avoid your asthma triggers?	Yes/No
		How to tell when your asthma is not well-controlled?	Yes/No

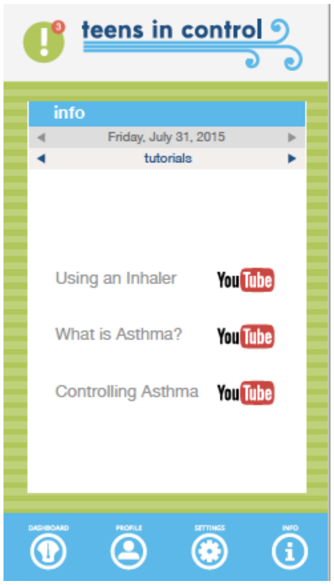

Games and quizzes	10	Do you like the idea of games?	Yes/No
		Do you like having badges or icons for achieving goals?	Yes/No
		How often would you play a game/quiz (not asthma control quiz)?	Open-ended

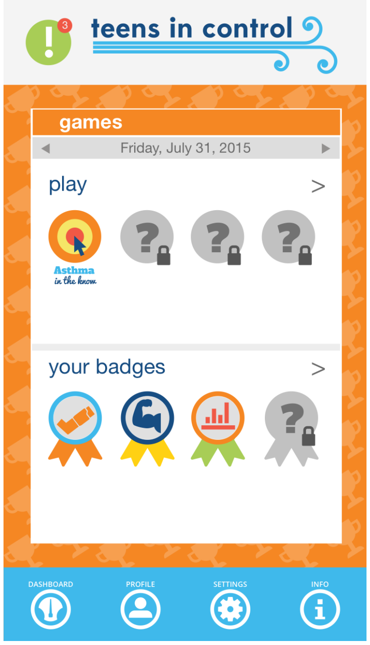

Asthma Control Quiz	5	Would you find an asthma control quiz useful?	Yes/No
		How often would you take this quiz?	Open-ended
		Do you like the smiley-face response options?	Yes/No

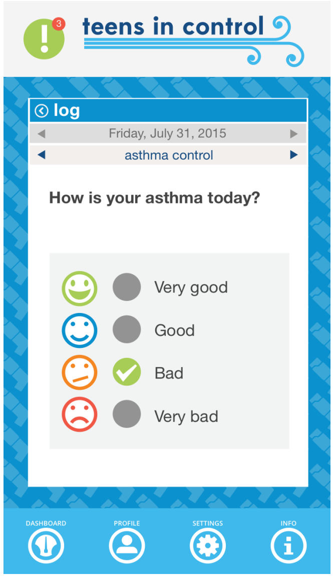

Logging Medications, Symptoms, and Triggers	19	Is there anything you like or dislike?<br>Anything you expected to do but couldn't?<br>Is there anything missing?<br>Is there anything you would do differently?	Open-ended<br>Open-ended<br><br>Open-ended<br>Open-ended

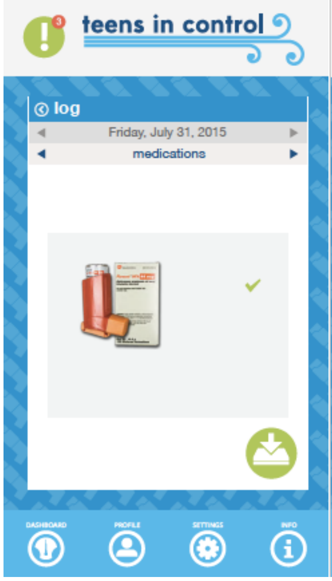

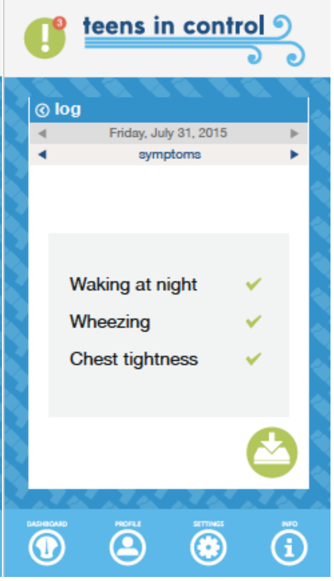

Charting	7	Anything you expected to do but couldn't?<br>Is there anything missing?<br>Is there anything you would do differently?<br>What do the dots/bar/line mean to you?	Open-ended<br>Open-ended<br>Open-ended<br>Open-ended

		How would you rate the asthma control of this person [for bar chart and line graph]?	4-point (4=very controlled, 3=controlled, 2=uncontrolled, 1=very uncontrolled)
		Which type of chart do you prefer?	Bar chart or line graph
		Mentions customization	Yes/No

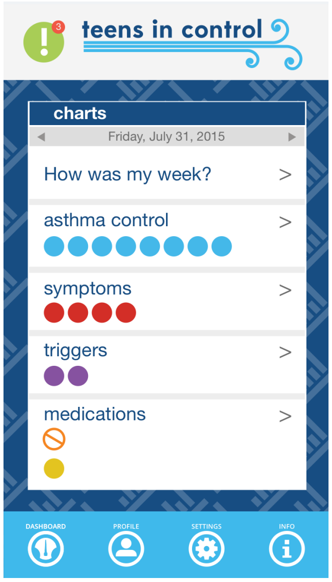

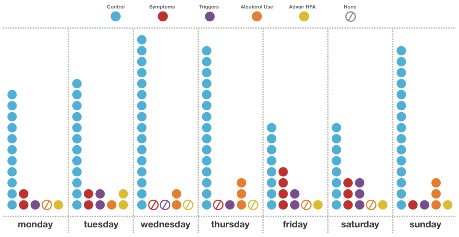

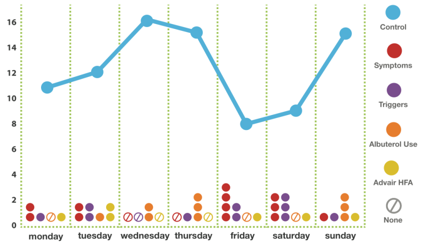

Notifications, reminders, and alerts	11	Would you use notifications/reminders/alerts for:	
		Triggers?	Yes/No
		Medications?	Yes/No

	Doctor's appointments?	Yes/No
	Anything else?	Yes/No
	How often would you use reminders?	Open-ended
	Mentions customization	Yes/No
	How would you like to be reminded for medications (e.g., every day, when a dose is missed)?	Open-ended

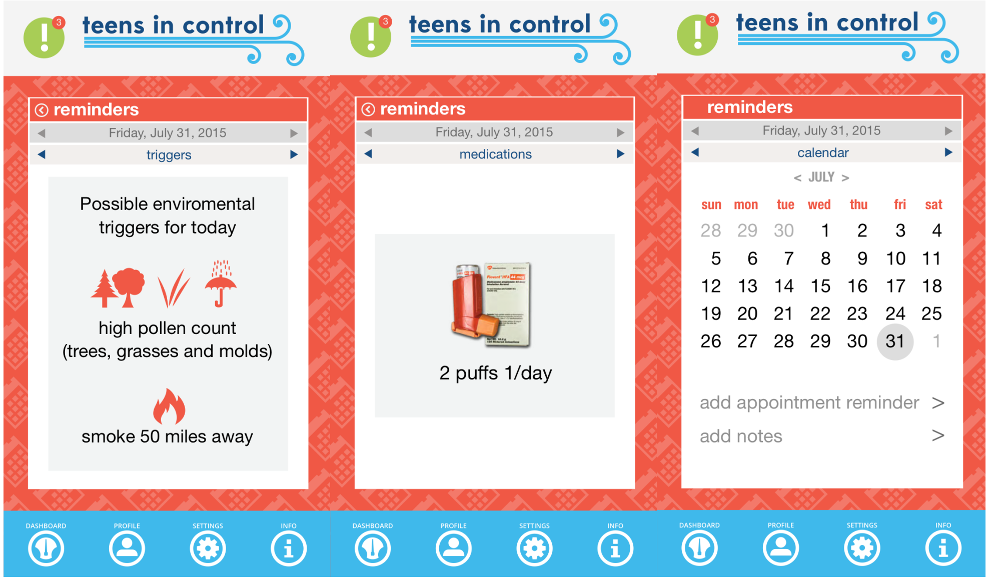

Summary Questions	Overall, what did you like about the app?	Open-ended
	Overall, what did you not like about the app?	Open-ended
	On a 1-5 scale, how likely would you be to use this app?	5-point scale (1 = not at all likely, and 5 = very likely)
	If changes (you suggested) were made, would you be more likely to use the app?	Yes/No
	Would you find an app like this useful?	Yes/No
	What are the top 3 things you liked about the app?	Open-ended
	What are the top 3 things you did not like about the app?	Open-ended
